# rhIGF-1 Therapy for Growth Failure and IGF-1 Deficiency in Congenital Disorder of Glycosylation Ia (*PMM2* Deficiency)

**DOI:** 10.1177/2324709613503316

**Published:** 2013-09-05

**Authors:** Bradley S. Miller, Meghann M. Duffy, O. Yaw Addo, Kyriakie Sarafoglou

**Affiliations:** 1 University of Minnesota Amplatz Children’s Hospital, Minneapolis, MN, USA

**Keywords:** growth, insulin-like growth factor-1 (IGF-1), glycosylation, congenital disorders of glycosylation

## Abstract

*Background*. Congenital disorders of glycosylation (CDG) are a group of rare disorders in which glycosylation required for proper protein-protein interactions and protein stability is disrupted, manifesting clinically with multiple system involvement and growth failure. The insulin-like growth factor (IGF) system plays an important role in childhood growth and has been shown to be dysfunctional with low IGF-1 levels in children with CDG type Ia (*PMM2* deficiency). *Case report*. A 3-year-old Caucasian male with failure to thrive was diagnosed with PMM2-CDG at 5 months of age. Initially, his length and weight were less than −2 standard deviation score, IGF-1 <25 ng/mL (normal 55-327 ng/mL), IGFBP-3 1.0 µg/mL (normal 0.7-3.6 ng/mL), and acid-labile subunit 1.3 mg/L (normal 0.7-7.9 mg/L). Despite aggressive feeding, he continued to show poor linear growth and weight gain. At 17 months, he underwent an IGF-1 generation test with growth hormone (0.1 mg/kg/d) for 7 days; baseline IGF-1of 27 ng/mL (normal 55-327 ng/mL) stimulated to only 33 ng/mL. Recombinant human IGF-1 (rhIGF-1) therapy (up to 130 µg/kg/dose twice daily) was initiated at 21 months of age resulting in an excellent linear growth response with height increasing from −2.73 to −1.39 standard deviation score over 22 months. IGF-1 and IGFBP-3 levels also increased. *Conclusion*. This is the first case report of rhIGF-1 therapy in a patient with PMM2-CDG. The child had an excellent linear growth response. These results provide additional in vivo evidence for IGF dysfunction in PMM2-CDG and suggest that rhIGF-1 may be a novel treatment for growth failure in PMM2-CDG.

## Introduction

Congenital disorders of glycosylation (CDG) are rare disorders of carbohydrate metabolism in which N-linked glycosylation is defective. These enzymatic defects lead to alterations in activity, structure, and stability of glycosylated glycoproteins. Glycoproteins are necessary for many cell processes, including protein transport, protein processing, cell signaling, protein–protein interactions, cell growth, immune system, and endocrine functions.^1-5^

PMM2-CDG (formerly CDG-Ia, OMIM 601785) is the most common CDG, resulting from a mutation in the *pmm2* gene that encodes phosphomannomutase, a cytosolic enzyme that converts mannose 6-phosphate to mannose-1-phosphate. Currently, no specific treatment exists to correct the glycosylation abnormalities in PMM2-CDG. Manifestations of PMM2-CDG are multisystemic and include psychomotor retardation, seizures, cardiomyopathy, coagulopathies, protein-losing enteropathy, liver disease, poor feeding, and failure to thrive.^6^ A combination of poor nutritional status and impairment of the growth hormone (GH)–insulin-like growth factor-1 (IGF-1) cascade is suggested as the cause of failure to thrive.^4,5^

Although not all processes in the GH-IGF-1 cascade are glycosylated, in vitro and in vivo evidence has demonstrated that defective glycosylation leads to lower levels of IGF-1, IGF-2, acid-labile subunit (ALS), and IGF binding protein-3 (IGFBP-3) that may contribute to poor linear growth in these patients.^5^ Inadequate glycosylation of ALS and IGFBP-3 leads to a reduction in the formation of the ternary complex necessary to stabilize IGF-1 and transport it to the target tissues because of increasing susceptibility to proteolysis (IGFBP-3) and reduced binding affinity (ALS).^7,8^

We describe the first PMM2-CDG patient to be treated with recombinant human IGF-1 (rhIGF-1) therapy who showed an excellent growth response.

## Materials and Methods

Clinical data were obtained by chart review of child with PMM2-CDG.

## Results/Case Report

We present a 3-year 7-month–old Caucasian male with PMM2-CDG who was born at $37\frac{2}{7}$ weeks gestation following an uncomplicated pregnancy. His birth weight was 2.95 kg (−1.01 standard deviation score [SDS]) and his birth length was 44.5 cm (−2.04 SDS). At 3.5 months of age, he had significant failure to thrive, esotropia, hepatomegaly, hypotonia with gross motor delays, inverted nipples, micropenis with palpable testes, and otherwise normal external genitalia and abnormal subcutaneous adipose tissue distribution. Ophthalmological evaluation was negative for retinitis pigmentosa. Laboratory evaluation showed elevated liver enzymes, low albumin (Table 1) due to protein losing enteropathy, thrombocytosis with a platelet count greater than 1 million and leukocytosis with a white blood cell count of 40.2 × 10^3^/mm^3^ (normal 6-17.5 × 10^3^/mm^3^) with no evidence of an infectious process, elevated international normalized ratio at 1.43 (normal 0.86-1.14), decreased antithrombin III at 24%, decreased protein C at 28%, and normal free protein S levels at 60%. He was also found to have enlarged nonpolycystic kidneys with normal renal function and no clinical evidence of proteinuria. Upper gastrointestinal endoscopy was normal and showed no intrahepatic biliary duct dilation.

**Table 1.** Auxologic and Biochemical Data.^a^

	Chronological Age								
	5 mo	9 mo	11 mo	1 y 2 mo	1 y 5 mo	1 y 9 mo	2 y 6 mo	3 y 1 mo	3 y 7 mo
Height (cm)	57.6	63.2	67.0	68.4	72.8	75.8	84	89.2	94
Height SDS	−3.36	−3.32	−3.05	−3.45	−2.69	−2.73	−1.86	−1.72	−1.39
Growth velocity (cm/y)		20.0	15.8	6.7	18.3	8.6	11.5	8.5	8.9
Growth velocity SDS		−0.44	+1.49	−2.45	+1.33	−0.96	+2.50	+0.36	+1.04
Weight (kg)	5.0	6.0	6.7	7.8	8.8	8.8	10.6	11.0	12.9
Weight SDS	−3.05	−3.75	−3.86	−3.09	−2.52	−3.11	−2.27	−2.67	−1.69
rhIGF-1 dose (µg/kg/dose)						40 to >70	120	130	130
IGF-1 (ng/mL)		<25	<25	31	27	64	39	53	346
IGF-1 SDS		Less than −2.5	Less than −2.5	−2.4	−2.5	−1.9	−2.2	−1.1	+2.9
IGFBP3 (µg/mL)		1.1	1.0		1.5	1.0	1.0	1.2	1.9
Albumin (U/L)	2.7		2.4	2.6		3.4	4.0	3.8	4.2
ALT (U/L)	147	194	101	124		229	41	33	31
AST (U/L)	103	128	166	133		336	68	54	45

Abbreviations: SDS, standard deviation score; IGF-1, insulin-like growth factor-1; ALT, alanine transaminase; AST, aspartate transaminase.

a Normal ranges: AST, 0-60 U/L; ALT, 0-50 U/L; albumin, 2.6-4.2 U/L (5 days to 12 months), 3.9-5.1 U/L (1-54 years); IGF-1, 55-327 ng/mL (1-23 months), 51-303 ng/mL (24-35 months), 49-289 ng/mL (36-47 months); IGFBP-3, 0.7-3.6 µg/mL (1-23 months), 0.8-3.9 µg/mL (24-35 months), 0.9-4.3 µg/mL (36-47 months).

Because of his clinical presentation, a carbohydrate-deficient transferrin analysis was requested (Mayo Medical Laboratories, Rochester, MN). Mono- and unglycosylated forms of transferrin were elevated (mono-oligosaccharide/di-oligosaccharide ratio = 3.796 (≤0.074) and Asialo oligosaccharide/di-oligosaccharide ratio = 2.619 (≤0.022) indicating a positive screen for CDG. Genetic testing (Greenwood Genetic Center, Greenwood, SC) demonstrated a G>C change at nucleotide 169 of the *pmm2* gene, which is predicted to result in a glycine being replaced by an arginine at amino acid 57 (p.G57R); this change has not been previously reported as a polymorphism or as a pathogenic change. The second nucleotide change G>A at nucleotide 422 is predicted to result in an arginine being replaced by a histidine at amino acid 147 (p.R141H) and it is a common pathogenic mutation in children with PMM2-CDG.^9^

**Figure 1.**
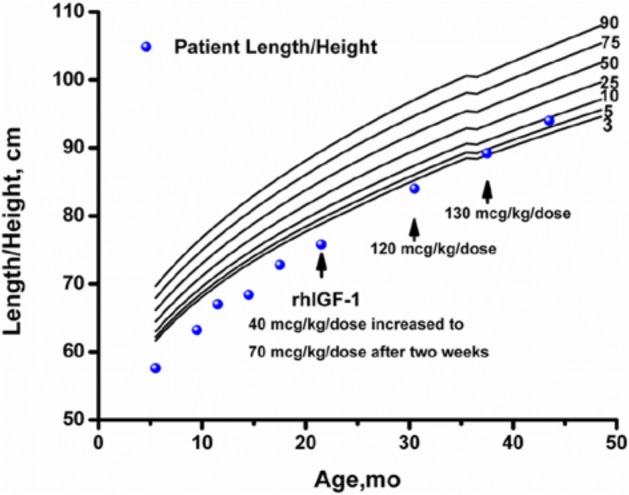
Growth curve of child with PMM2-CDG (PMM2 congenital disorder of glycosylation) treated with rhIGF-1. Arrow indicates time at which each rhIGF-1 dose was initiated.

**Figure 2.**
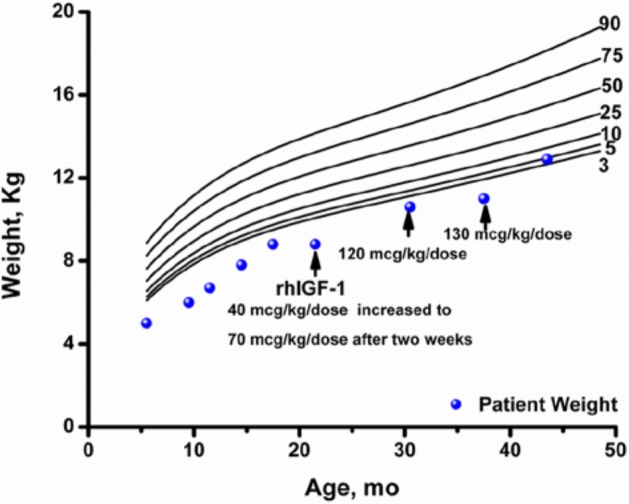
Weight of child with PMM2-CDG (PMM2 congenital disorder of glycosylation) treated with rhIGF-1. Arrow indicates time at which each rhIGF-1 dose was initiated.

Despite an aggressive feeding schedule with overnight gastrostomy tube supplementation he showed continued growth failure (height −2.69 SDS, weight −2.52 SDS based on Centers for Disease Control and Prevention 2000 growth charts,^10^; Figures 1 and 2) and low or undetectable IGF-1 levels (Table 1). At 17 months, he underwent an IGF-1 generation test with growth hormone (0.1 mg/kg/d) for 7 days. There was minimal IGF-1 (baseline 27 ng/mL, stimulated 33 ng/mL) and IGFBP-3 (baseline 1.5 µg/mL, stimulated 1.3 µg/mL) generation response to this high-dose rhGH therapy. An increase in IGF-1 of <15 ng/mL during an IGF-1 generation test is consistent with growth hormone resistance while normal individuals demonstrate an increase of >300 ng/mL. rhIGF-1 therapy was started at 21 months at 40 µg/kg/dose twice daily for 2 weeks, then increased to 70 µg/kg/dose twice daily. The rhIGF-1 dose was increased to the target dose of 120 µg/kg/dose twice daily at 30 months of age. The rhIGF-1 dose was increased to 130 µg/kg/dose twice daily at 37 months of age because of slowing of the growth velocity. An IGF-1 level was obtained 2 to 4 hours after the rhIGF-1 dose at 43 months of age to assess peak response with goal being <+4 SDS. While on rhIGF-1 therapy, height SDS score consistently improved, going from −2.73 at initiation of therapy to −1.39 after 21 months of therapy. Serum levels of IGF-1 were low at initiation of therapy and improved with rhIGF-1 therapy. IGFBP-3 levels were normal prior to and during rhIGF-1 therapy.

Because of the risk of hypoglycemia with rhIGF-1 therapy, at the start of therapy the child’s mother was instructed to measure blood glucose before and 2 hours after each dose. During rhIGF-1 therapy, the family documented a single episode of asymptomatic hypoglycemia (glucometer reading “in the low 60s”) associated with a febrile illness.

Aspartate transaminase and alanine transaminase, which were elevated prior to therapy, normalized at 3.5 years of age. Protein-losing enteropathy improved with normal albumin levels at 2.5 years of age (Table 1).

## Discussion

Failure to thrive is an important clinical feature of PMM2-CDG and is thought to be caused by a combination of poor nutritional status and impairment of the GH-IGF-1 cascade.^4,5^ Children with PMM2-CDG have been shown to have significantly decreased levels of ALS, IGFBP3, IGF-1 and IGF-2, and ternary complex formation compared with age-matched controls.^5^ The key role of GH-IGF system glycosylation in the impaired growth of children with CDG is demonstrated by the growth response of a child with PMI-CDG (formerly CDG Ib, OMIM 602579) who received mannose therapy to bypass the enzymatic defect. Prior to mannose therapy, she was obese due to enteral nutrient therapy of hypoglycemia. During mannose therapy, she showed excellent catch-up in linear growth accompanied by improvement in the glycosylation of ALS and IGFBP-3 despite a reduction in overall calories.^5^ This child’s growth response suggests that improvement of glycosylation in the GH-IGF cascade led to catch-up growth independent of nutritional status.

Because of the rarity of the condition, data on long-term linear growth in children with CDG are limited.^11,12)^ In the largest longitudinal study of children with PMM2-CDG, significant failure of weight gain and linear growth was seen in the first 6 to 9 months of life (mean weight −3 SDS; mean length −2 SDS followed by a slight increase to a maximum of −1.8 SDS at the end of the second year of life. After age 2 years, the mean length/height SDS decreased again and persisted without improvement in weight or height by 10 years of age.^12^ Analysis of the GH-IGF system in children with PMM2-CDG is also limited. Elevated basal and glucagon-stimulated GH levels have been shown in some females with PMM2-CDG.^13^ However, basal GH levels have been shown to be normal in some males.

We report on the first PMM2-CDG child with severe failure of growth and weight gain and low or undetectable levels of IGF-1 treated with rhIGF-1. The child’s small increase in IGF-1 and lack of increase in IGFBP-3 in response to the IGF-1 generation test (0.1 mg/kg/d rhGH for 7 days) suggested GH resistance rather than GH deficiency. GH resistance in this patient could be because of abnormalities of the GH receptor or postreceptor signaling pathway or because of a shortened half-life of IGF-1 stemming from the instability of the ternary complex components, ALS and IGFBP-3. The excellent linear growth response to rhIGF-1 therapy indicates that IGF-1 deficiency is a key component of growth failure in PMM2-CDG. Because of the complexity of the effect of deficient glycosylation on the GH-IGF system,^4,5^ the mechanism of IGF-1 deficiency in these patients could be multifactorial stemming from varying degrees of GH resistance, liver dysfunction,^14^ and inadequate nutrition/malabsorption. Nonetheless, as our report shows, rhIGF-1 therapy has the potential to improve linear growth in children with PMM2-CDG.

Hepatopathy and elevated transaminases are well-documented findings in children with PMM2-CDG.^9,15-18^ Our patient with PMM2-CDG showed evidence of hepatopathy (elevated transaminases) and protein-losing enteropathy (low albumin), which normalized while on rhIGF-1 treatment. Since the typical course is normalization of the transaminases within the first decade of life,^9,15,16^ it is unclear if IGF-1 therapy played a role through cell growth promotion, wound healing and barrier function in the intestine.^19,20^

The clinical improvement of growth of this child with PMM2-CDG following rhIGF-1 therapy provides further evidence for the importance of glycosylation in the GH-IGF cascade and suggests that rhIGF-1 may be a novel treatment for growth failure in PMM2-CDG reversing the loss of growth potential that has been observed during the first 2 years of life.^12^ Further study of the role of rhIGF-1 therapy in CDG is warranted.
